# Reducing Dietary Acrylamide Exposure from Wheat Products through Crop Management and Imaging

**DOI:** 10.1021/acs.jafc.2c07208

**Published:** 2023-02-06

**Authors:** Joseph Oddy, John Addy, Andrew Mead, Chris Hall, Chris Mackay, Tom Ashfield, Faye McDiarmid, Tanya Y. Curtis, Sarah Raffan, Mark Wilkinson, J. Stephen Elmore, Nicholas Cryer, Isabel Moreira de Almeida, Nigel G. Halford

**Affiliations:** † Rothamsted Research, Harpenden, Hertfordshire AL5 2JQ, United Kingdom; ‡ Crop Health and Protection (CHAP), Rothamsted, Harpenden AL5 2JQ, United Kingdom; § Curtis Analytics Limited, Discovery Park, Sandwich CT13 9FE, United Kingdom; ∥ Department of Food and Nutritional Sciences, University of Reading, Reading RG6 6DZ, U.K.; ⊥ Mondele̅z UK R&D Ltd, Bournville Lane, Bournville, Birmingham, B30 2LU, U.K.; # Mondele̅z R&D International, 6 Rue René Razel, Saclay 91400, France

**Keywords:** acrylamide, asparagine, biscuits, food safety, multispectral imaging, nitrogen, phosphorus, potassium, sulfur, wheat

## Abstract

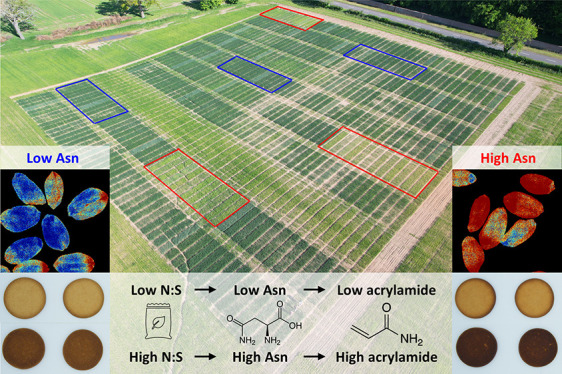

The nutritional safety of wheat-based food products is compromised by the presence of the processing contaminant acrylamide. Reduction of the key acrylamide precursor, free (soluble, non-protein) asparagine, in wheat grain can be achieved through crop management strategies, but such strategies have not been fully developed. We ran two field trials with 12 soft (biscuit) wheat varieties and different nitrogen, sulfur, potassium, and phosphorus fertilizer combinations. Our results indicated that a nitrogen-to-sulfur ratio of 10:1 kg/ha was sufficient to prevent large increases in free asparagine, whereas withholding potassium or phosphorus alone did not cause increases in free asparagine when sulfur was applied. Multispectral measurements of plants in the field were able to predict the free asparagine content of grain with an accuracy of 71%, while a combination of multispectral, fluorescence, and morphological measurements of seeds could distinguish high free asparagine grain from low free asparagine grain with an accuracy of 86%. The acrylamide content of biscuits correlated strongly with free asparagine content and with color measurements, indicating that agronomic strategies to decrease free asparagine would be effective and that quality control checks based on product color could eliminate high acrylamide biscuit products.

## Introduction

Since large-scale industrial manufacturing of biscuits and cakes started in the 1800s, these foods have become staple items in the food culture of many parts of the world.^1^ In 2020, participants of the UK National Diet and Nutrition Survey recorded a consumption of 20 g of biscuits and 16.75 g of buns, cakes, and pastries each day (averaged across all age groups).^2^ This was reflected by the 2.96 billion GBP in UK biscuit sales in 2020 and by the estimate that 99.5% of all households purchased biscuits in 2020.^3^ Consequently, there is a large market in the UK for soft milling wheat flour, with UK flour millers producing an average of 541,000 tons of biscuit flour and 81,000 tons of cake flour annually from 1991 to 2020.^4^ In the USA, approximately 9.82 million tons of soft red winter wheat production are forecast for the year 2021/2022,^5^ providing flour for a biscuit market worth approximately 11.7 billion USD in 2021.^6^

Soft milling wheats (UK Flour Millers group 3) are the primary crop used in the baking of biscuits, cakes, breakfast cereals, and fine bakery products because they have lower protein content than hard, breadmaking wheats (11–11.5% protein content requirement for soft wheats vs 13% requirement for breadmaking) and have a soft endosperm texture.^7^ Soft wheat grains are easily fractured, so they exhibit less starch damage and less water absorption during milling and processing than hard wheat grains.^8^ Due to their lower protein requirement, group 3 wheats do not require as much nitrogen fertilizer as groups 1 (breadmaking) or 2 (breadmaking potential) wheats.^9^ At the time of writing, there are 10 group 3 varieties on the 2022/2023 UK winter wheat recommended list but only four group 1 and four group 2 varieties.^10^ These factors may drive an increase in soft wheat cultivation, but increasing prices obtainable for breadmaking wheat, the soaring cost of nitrogen fertilizer, and many other factors will also affect farmers’ decision-making.^11^

Another factor that farmers are increasingly having to manage is the acrylamide-forming potential of their wheat. Acrylamide is a “probably carcinogenic”, neurotoxic, and reproductively toxic contaminant that forms from free asparagine and reducing sugars (principally glucose, fructose, and maltose) in the Maillard reaction during frying, baking, roasting, toasting, and high-temperature processing.^12,13^ Other amino acids also participate in the Maillard reaction, giving rise to the color and flavor compounds that impart fried, baked, roasted, and toasted products with their signature characteristics. Dietary exposure to acrylamide is considered to be a public health risk by the European Food Safety Authority,^14,15^ prompting the European Commission to introduce a series of risk management measures, most recently Commission Regulation (EU) 2017/2158,^16^ which came into force in 2018. Commission Regulation (EU) 2017/2158 sets benchmark levels (described by the Commission as performance indicators) for acrylamide in different food categories. These included 50 μg/kg for soft bread, 350 μg/kg for biscuits (150 μg/kg if they are for infants), 400 μg/kg for crackers, 300 μg/kg for wheat-based breakfast cereals, 150 μg/kg for breakfast cereals made with other grains, and 40 μg/kg for cereal-based baby foods.

Based on estimates of dietary acrylamide intake from EFSA,^14,17^ soft wheat products (biscuits, crackers, breakfast cereals, crispbreads, cakes, and pastries) are major contributors to dietary acrylamide intake, even more so than bread if taken together. Consequently, biscuit, breakfast cereal, crispbread, cake, and pastry manufacturers must minimize the concentrations of acrylamide in their products as much as possible, and various strategies for doing so have been compiled in FoodDrinkEurope’s “Acrylamide Toolbox”.^18^ However, while reducing acrylamide formation, food businesses must avoid impacting flavor, aroma, texture, and color and ending up with a bland, insipid product that consumers reject.

A factor that makes it more difficult for food businesses to keep the acrylamide levels in their products consistently below the benchmark level is the highly variable and unpredictable concentrations of free asparagine and reducing sugars in the raw materials they use. For example, average potato chip (UK crisp) acrylamide levels in Europe have declined substantially since acrylamide was discovered in food in 2002 and mitigation strategies began to be introduced, with European Snacks Association data showing a reduction of 54% between 2002 and 2019.^19^ Nevertheless, in the three-year period from 2017 to 2019, 7.75% of potato chip samples still failed the 750 μg/kg benchmark level, and seasonal and geographical factors exacerbated the problem, with almost 18% of samples in southern Europe in January and above 10% in every region for some of the year exceeding the benchmark level. Similarly, a recent study in Spain^20^ found that 15% of breakfast cereals contained acrylamide above the benchmark level. It is, therefore, important to act on this issue because the European Commission is considering replacing benchmark levels with maximum levels (i.e., levels above which it would be illegal to sell a product) and look likely to set maximum levels at or close to the current benchmark levels.^21^

For soft milling wheat products, it is the environmental impact on free asparagine concentration in the grain that poses the largest risk for acrylamide formation as free asparagine is the key determinant of acrylamide formation in wheat products.^17^ Unfortunately, free asparagine is difficult and expensive to measure, usually requiring HPLC or GC/LC–MS methods for quantification.^17^ Enzymatic methods for free asparagine detection remove some of this complexity and cost,^22^ but they still require multistep sample preparation. Measurement of grain protein content can be achieved rapidly and non-destructively using near infrared spectroscopy (NIRS),^23^ but similar attempts to use NIRS to measure free asparagine in wheat grain have found low predictive ability.^24^

Both abiotic and biotic stressors are known to increase free asparagine in the grain,^25^ so certain crop management strategies are included in the compulsory mitigation measures set out in Commission Regulation (EU) 2017/2158. These include avoiding excessive nitrogen (N) application while ensuring adequate sulfur (S) supply. However, there is still uncertainty about the optimal levels of N and S *per se* that should be applied and the effect of the N:S ratio. Additionally, the impact of other minerals (phosphorus and potassium) on free asparagine concentrations is not known. Consequently, this study aimed to investigate these uncertainties, encompassing not only the effects of fertilization on free asparagine concentration but also the impacts on biscuit quality and acrylamide concentration after baking. We also investigated whether free asparagine could be predicted from multispectral measurements of plants growing in the field and from seeds.

## Materials and Methods

### Screening and Selection of Soft Wheat Varieties

DNA was extracted from a selection of soft wheat varieties and screened for the presence of the *ASN-B2* gene (TraesLDM3B03G01566640 in variety Landmark; Ensembl, 2021) as described previously.^26^ Varieties lacking *ASN-B2* were then used in this study, comprising Arkeos (2010, Limagrain), Barrel (2014, KWS), Basset (2015, KWS), Claire (1997, Limagrain), Croft (2012, KWS), Elicit (2017, Elsoms Wheat), Firefly (2017, KWS), Horatio (2011, Limagrain), Invicta (2008, Limagrain), Leeds (2011, KWS), Myriad (2011, Limagrain), and Zulu (2012, Limagrain). Data on variety registration dates and breeding companies were obtained from the EU plant variety database^27^ and UK national lists.^28^ Claire was used as a negative control and Cadenza as a positive control when screening for the presence of *ASN-B2* due to the availability of these genomes in Ensembl Plants.^29^ The results of this screen are displayed in Supplementary Figure 1.

### Field Trials

Field trials were undertaken at two different locations across the Rothamsted Research experimental farm site at Woburn: Stackyard (51° 59′ 53.3832″ N 0° 37′ 1.3008″ W) in 2019/2020 (H20) and Butt Clong (52° 0′ 43.7184″ N 0° 35′ 45.5388″ W) in 2020/2021 (H21) (Supplementary Figure 2). Key dates for these trials are given in Supplementary Table 1. The trial at Stackyard was undertaken using treatments A to K listed in Table 1, whereas the trial at Butt Clong used treatments A to L. The size of experimental plots in each trial was 9 × 1.8 m. Stackyard has a loamy sand to sandy loam soil, whereas Butt Clong has a sand to loamy sand soil.^30^ See Supplementary Figure 3 for the layout of each trial.

**Table 1:** Fertilizer Treatments Applied in this Study (Application Rates Given in Kilograms per Hectare)

treatment	nitrogen	sulfur	phosphorus	potassium
A	200	40	35	62
B	200	20	35	62
C	200	10	35	62
D	200	0	35	62
E	100	40	35	62
F	100	20	35	62
G	100	10	35	62
H	100	0	35	62
I	100	20	35	0
J	100	20	0	62
K	100	20	0	0
L	200	0	0	0

Nitrogen (N) and sulfur (S) were applied as DoubleTop (CF fertilizers) (ammonium sulfate and ammonium nitrate mixture, 27 N (30SO_3_)). Nitram (CF fertilizers) (ammonium nitrate, NH_4_NO_3_) was applied for sulfur-deficient plots and to supplement the DoubleTop application where necessary to reach the required nitrogen treatment rates. Phosphorus was applied as triple super phosphate (TSP) (Diamond Fertilizers) (P_2_O_5_) and potassium was applied as muriate of potash (MOP) (Diamond Fertilizers) (K_2_O). See Supplementary File 2 for further details of fertilizer treatments.

At Stackyard, varieties were drilled at a rate of 350 seeds/m^2^ except for Croft (343 seeds/m^2^), Invicta (376 seeds/m^2^), and Leeds (289 seeds/m^2^) due to differences in germination, seed damage, and seed availability. Due to a problem with drilling, plot 11 was smaller than the rest and reliable yield measurements could not be taken. Sulfur, potassium, and phosphorus were applied at the same time as the first nitrogen split in this trial (10/03/2020). Herbicide was sprayed as a mixture of Palio (Corteva), Cogent (Intracrop), and Sprinter (Nufarm) at a rate of 0.265 kg/ha on 24/03/2020 to control weeds.

At Butt Clong, all varieties were drilled at a rate of 350 seeds/m^2^. Only Nitram was applied during the first split (23/02/2021), with the other fertilizers being applied on 11/04/2021. Pesticide was sprayed as a mixture of Samurai (Bayer CropScience) (3 L/ha) and Buffalo Elite (Intracrop) (1 L/ha) on 24/06/2021. Plots 242 and 295 were mixed during harvest, preventing further analysis of these plots, but this did not affect yield measurements.

Weather measurement data were retrieved from the Rothamsted electronic archive resource,^31^ which contains daily data from a weather station at the Woburn experimental field site. Daily temperature, rainfall, and solar radiation measurements over the periods that both trials were grown are shown in Supplementary Figure 4.

### Grain Sample Preparation and Amino Acid Analysis

After harvest, a sub-sample of each plot was weighed to calculate fresh weight. This sub-sample was then oven-dried at 80 °C for 24 h and then reweighed to measure the dry weight. The percentage reduction in weight from lost moisture content was then used to adjust yield estimates taken at harvest on the combine and to calculate grain yield at 85% dry matter. For long-term storage, grain was oven-dried to reduce moisture content to between 8 and 10%. Thousand grain weight (TGW) measurements were subsequently taken by counting 500 seeds using a seed counter (Elmor model C1, Switzerland), drying overnight at 80 °C, and then weighing. This was repeated twice to give TGW measurements.

For samples from the first trial at Stackyard, approximately 80 g from each plot were milled to fine wholemeal flour using a Retsch 400 ultra-centrifugal mill (Retsch GmbH, Germany). Samples of grain from Butt Clong were milled to wholemeal flour in a coffee grinder. Flour moisture content was determined using a Minispec nuclear magnetic resonance analyzer (Minispec Mq10, Bruker Inc., Germany). Following determination of moisture content, the Hagberg falling number was measured using an FN 1000 (Perten, Sweden). Free asparagine analysis (measured as mmol per kg) was performed on wholemeal flour samples by HPLC as described previously^32^ by Curtis Analytics (Sandwich, UK). Briefly, this entailed extraction of free amino acids with hydrochloric acid and subsequent derivatization with *o*-phthalaldehyde. Samples were then measured by HPLC alongside a series of standards for quantification.

### Multispectral Phenotyping and Grain Imaging Analysis

Multispectral measurements were taken in the field at Butt Clong using a Tec5 HandySpec Field spectrometer (Oberursel, Germany) as described previously.^33^ Measurements were taken for all 432 plots on six different dates from the 17th of May 2021 to the 6th of August 2021. Reflectance values were obtained for 65 wavelengths at 10 nm intervals between 360 and 1000 nm. NDVI_680_ (normalized difference vegetation index) and PSRI (plant senescence reflectance index)^34^ were calculated as previously described from wavelengths as shown below:
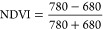

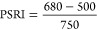


Grain samples from 72 plots at Butt Clong (all three replicates of all 12 varieties for the treatments N100 S10 + P + K and N200 S0 – P – K) were further analyzed using the Videometer SeedLab system (Videometer, DK) available in the Crop Health and Protection (UK)’s Digital Phenotyping Laboratory. This automated grain/seed imaging system can be used to determine reflectance values for 19 wavelengths ranging from 365 to 970 nm and fluorescence values by the optional use of four long-pass filters (400, 500, 600, and 700 nm cutoffs). In total, 70 features were calculated from the image data; 19 reflectance values, 31 fluorescence bands, and 20 morphological and color-based features (see Supplementary File 3). The seed and chaff were separated from background pixels and one another using custom classifiers developed using the Videometer SeedLab system. Only data from pixels classified as seeds were used in further analysis. See Supplementary File 3 for the data obtained from this analysis.

### Baking Tests

In order to perform our desired baking tests, we had to mill grain to white flour instead of wholemeal flour. Grain samples of variety Basset from the second trial at Butt Clong were milled to white flour using a Bühler mill by Campden BRI (Chipping Campden, UK), with an average extraction rate of 75.93% ± 0.42% (95% confidence intervals given by the ± symbol). Specifically, all samples of variety Basset from treatments G, H, and L (Table 1) from the second trial were selected as these samples showed a wide range of grain asparagine content. This flour was used to bake biscuits according to a modified AACC 10-53.01 protocol (Baking Quality of Cookie Flour: Macro Wire-Cut Formulation). Flour moisture content was measured using an HG63 Halogen Moisture Analyzer (Mettler Toledo), and the mean flour moisture content was 13.51% ± 0.23%. In order to achieve an equivalent 225 g flour weight at 13% moisture basis across baking tests, water and flour volumes were correspondingly increased or decreased.

To form the creamed mass, non-fat dry milk, salt, sodium bicarbonate, sugar, and palm oil were mixed in a Hobart N50 mixer for 3 min at speed 1 (136 rpm), stopping and scraping the contents every minute. The creamed mass was subsequently mixed with a solution of ammonium bicarbonate and high-fructose corn syrup (42%) in distilled water at speed 1 for 1 min and at speed 2 (281 rpm) for a further minute. Finally, the flour was mixed in to form the dough at speed 1 for 2 min, stopping and scraping contents every 30 s.

Dough was then rolled and cut into 4 × 5 cm portions on a single aluminum tray. Dough water activity was measured using a 4TE water activity meter (Aqualab), with mean water activity of 0.80 ± 0.01, and dough weight was measured before baking, with a mean weight of 101.0 g ± 1.2 g. Biscuits were baked for 11 min in a five-chamber Polin Elettrodrago oven at 205 °C and left to cool for 5 min on the tray outside the oven followed by a 45 min cooling period on a wire rack before storing in air-tight containers. This protocol was repeated twice for each flour sample.

### Color Measurements of Biscuits and Acrylamide Analysis

Biscuit diameter, stack height, and weight measurements were taken as the mean of all four biscuits from each half-batch. For color analysis, images of both the top and bottom of the biscuits were taken inside an LED light box (SAMTIAN) with a color temperature of 5500 Kelvin and a FinePix S8000fd digital camera (Fujifilm). Images were captured at a shutter speed of 1/250 s, an aperture size of *f*/4, and an ISO of 100. The biscuit pixel area was segmented from background pixels using the Simple Interactive Object Extraction plugin in Fiji. RGB images were then converted to Lab Stack images, and mean values for CIELAB color space parameters (L*, a*, and b*) were taken from the total area of all biscuits in an image.

One biscuit was taken from each half-batch for acrylamide analysis at Reading Scientific Services Ltd. (UK), with one technical replicate being taken from each sample. Each biscuit was ground in a coffee grinder, and approximately 1.0 g (± 0.1 g) of each sample was used for further analysis. Extraction was then performed with an internal standard (D_3_-acrylamide solution) and MQ water at 60 °C (± 5 °C). Precipitation was subsequently performed using Carrez reagents, and ethyl acetate was then added to perform liquid–liquid extraction of the supernatant. The clear supernatant was then evaporated, and the acrylamide was purified by solid-phase extraction. Liquid chromatography with tandem mass spectrometry was then used with acrylamide standards to measure the acrylamide content of the samples.

### Statistical Design and Analyses

Both field trials were designed as split-plot designs with three replicate blocks, with nutrient treatments applied to main plots (each comprising a linear array of 12 sub-plots) and varieties applied to sub-plots. Given the overall size of each experiment and the potential impact of farm operations, the allocation of varieties followed an incomplete (first trial) 11-by-12 Latin square design or complete (second trial) 12-by-12 Latin square design for each of the three sets of 11 or 12 main plots arranged down the length of the trial: this was to ensure that varieties were spread evenly across the width of each trial and therefore reduce any bias due to spatial variability (Supplementary Figure 3). However, this additional blocking structure was not incorporated into the analysis model, with sub-plots just assumed to be nested within main plots. For free asparagine, three technical replicates were collected from each sub-plot. A row of discard plots was incorporated into the design of the second trial to account for where an old hedgerow used to be in the field. Both field trials were designed using GenStat.^35^

Grain asparagine content was log_e_ transformed to account for non-normality and improve heteroscedasticity because it was positively skewed. Analysis of variance (ANOVA) was used to investigate the effects of different experimental factors while accounting for the structure of the trials. For visualization of results, least significant differences were calculated at 5% to plot alongside means from each model. Graphs were plotted in R^36^ with data manipulation using package “gdata”^37^ and in Genstat. Details of each model (treatment structure, blocking structure, and full ANOVA tables) are available in Supplementary Files 4 and 5 along with a description of the data filtering used in each analysis. Data used in these analyses are available in Supplementary File 6. Some of the analyses considered data from just a subset of the varieties, whereas other analyses considered the combined data from both trials. For the combined analysis, we included terms to test for the consistency of treatments between the two trials (the trial-by-treatment interactions).

For analysis of multispectral field data via partial least squares regression (PLSR), all wavelength measurements across all timepoints were combined (including NDVI and PDRI measurements) to form the predictor variables. Log_e_-transformed grain asparagine content and non-transformed yield measurements were used as the response variables. Based on the mean square error and *R*^2^ plots investigating the optimal number of components to include for each trait, three components were retained for the yield PLSR model and 10 components were retained for the asparagine PLSR model. Five-fold cross validation repeated 1000 times was used to test each model and collect *R*^2^ estimates. PLSR and plotting of multispectral data was performed using python and python packages NumPy,^38^ pandas,^39^ Scikit-learn,^40^ and plotnine. Data used for analysis are available in Supplementary File 7.

For analysis of data obtained from the Videometer SeedLab, measurements were obtained for a minimum of 200 seeds for each sample, and the mean was calculated for each variable to obtain a single measurement for each variable from each sample. Principal component analysis and linear discriminant analysis were performed and visualized in R with the packages factoextra,^41^ MASS,^42^ ggplot2,^43^ and cowplot.^44^ Correlation matrices were used for both principal component analyses performed in this study using the function “prcomp” and option “scale = TRUE” to account for different scales of measurement between variables. Gaussian naïve Bayes classification was performed and visualized using python and the same packages as described for PLSR above. Five-fold cross validation repeated 1000 times was also used to test the balanced accuracy of this model.

Analysis and visualization of biscuit data was performed in R with the packages factoextra,^41^ ggplot2,^43^ and cowplot.^44^ Data used in these analyses are available in Supplementary File 8. A hue angle (a color appearance parameter) was calculated using the below formula:
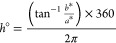


## Results

### Impact of Environment, Fertilizers, and Variety on the Free Asparagine Content of Wheat Grain

**Figure 1:**
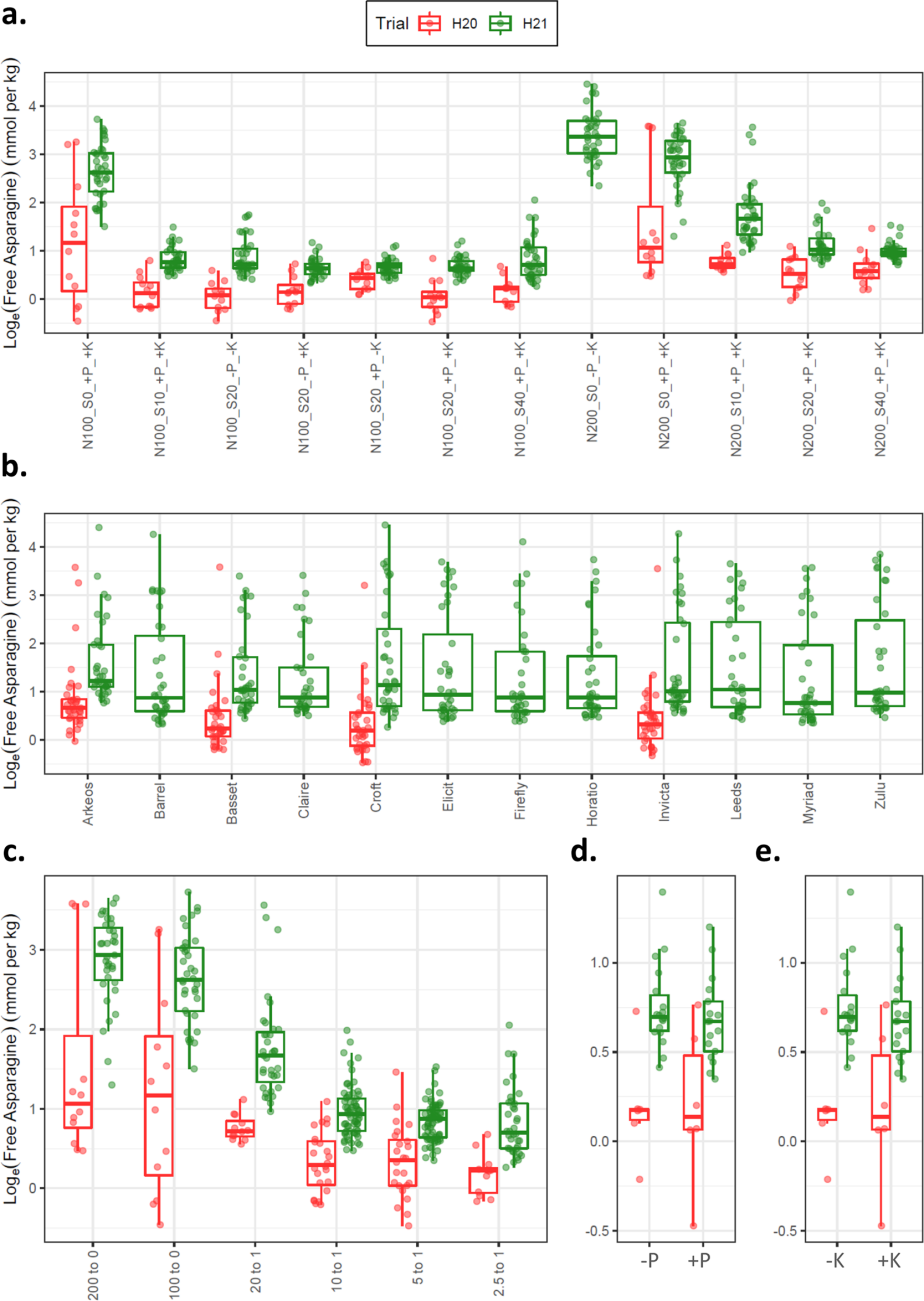
Free asparagine measurements in grain from both field trials. (a) Free asparagine measurements separated by agronomic treatment. (b) Free asparagine measurements separated by variety. (c) Free asparagine measurements separated by the nitrogen to sulfur ratio. (d) Free asparagine measurements separated by phosphorus treatment. (e) Free asparagine measurements separated by potassium treatment. Boxes show first and third quartiles alongside the median. Whiskers extend to the largest data points within 1.5 times the interquartile range. H20 (2019/2020 trial) and H21 (2020/2021 trial). −P (0 kg/ha phosphorus), −K (0 kg/ha potassium), +P (35 kg/ha phosphorus), and +K (62 kg/ha potassium).

In order to analyze the impact of environment, fertilizer treatment, variety, and the interaction between these factors on the free asparagine content of wheat grain, we constructed ANOVA models investigating the overall impact of treatment (Table 2), the N:S ratio (Table 3), and the application of P and/or K (Table 4). Full details of the data used for each analysis and the modeling terms are provided in Supplementary Files 4 and 5, in addition to an analysis of nitrogen and sulfur as interacting terms (Supplementary Table 2 and Supplementary File 5 and our analyses of yield. These analyses showed that treatment significantly impacted the free asparagine content of the grain across both trials (Table 2, Figure 1a, and Supplementary Figure 6c,d) and that this was principally due to the N:S ratio (Table 3, Figure 1c, Supplementary Figure 7a), whereas potassium and phosphorus did not significantly impact grain asparagine content in either trial (Table 4, Figure 1d,e, Supplementary Figure 7b,c). Variety did significantly impact the free asparagine content of the grain across both trials; however, the differences between varieties were not as great as those between trials or treatments (Figure 1b and Supplementary Figure 6a,b). Non-transformed free asparagine data are shown in the form of heatmaps in Supplementary Figure 5.

**Table 2:** Significance Values (*F* Probabilities) of Terms in ANOVA Models for Analysis of Log_e_-Transformed Free Asparagine Content in Grain (H20 (2019/2020 Trial) and H21 (2020/2021 Trial))

source of variation	H20	H21	both (nested)	both (full)
treatment	0.030	<0.001	<0.001	<0.001
variety	<0.001	<0.001	<0.001	<0.001
treatment × variety	0.183	0.005	0.004	0.119
trial	NA	NA	NA	0.006
trial × treatment	NA	NA	NA	0.524
trial × variety	NA	NA	NA	<0.001
trial × treatment × variety	NA	NA	NA	0.056

**Table 3:** Significance Values (*F* Probabilities) of Terms in N:S Ratio ANOVA Models for Analysis of Log_e_-Transformed Free Asparagine Content in Grain (H20 (2019/2020 Trial) and H21 (2020/2021 Trial))

source of variation	H20	H21	both
N:S ratio	0.050	<0.001	<0.001
variety	<0.001	<0.001	<0.001
N:S ratio × variety	0.024	<0.001	0.034
trial	NA	NA	0.012
trial × N:S ratio	NA	NA	0.471
trial × variety	NA	NA	<0.001
trial × N:S ratio × variety	NA	NA	0.009

**Table 4:** Significance Values (*F* Probabilities) of Terms in Phosphorus/Potassium ANOVA Models for Analysis of Log_e_-Transformed Free Asparagine Content in Grain (H20 (2019/2020 Trial) and H21 (2020/2021 Trial))

source of variation	H20	H21	both
phosphorus	0.353	0.413	0.700
potassium	0.321	0.353	0.195
phosphorus × potassium	0.087	0.284	0.381
variety	<0.001	<0.001	<0.001
phosphorus × variety	0.898	0.295	0.400
potassium × variety	0.498	0.723	0.572
phosphorus × potassium × variety	0.540	0.688	0.335
trial	NA	NA	<0.001
trial × phosphorus	NA	NA	0.244
trial × potassium	NA	NA	0.719
trial × phosphorus × potassium	NA	NA	0.035
trial × variety	NA	NA	0.094
trial × phosphorus × variety	NA	NA	0.583
trial × potassium × variety	NA	NA	0.810
trial × phosphorus × potassium × variety	NA	NA	0.872

There was a significant interaction between the variety and trial (Tables 2 and 3), indicating that the free asparagine content in the grain was not consistent for the varieties across environments. For example, Croft grain had the lowest mean free asparagine content in the 2020 harvest (H20), but the highest in the 2021 harvest (H21) (Figure 1b and Supplementary Figure 6a,b). In contrast, there was no interaction between the N:S ratio and trial (Table 3), suggesting that the N:S ratio had the same effect across environments. This can be seen in Figure 1c, where free asparagine content of the grain decreases in both environments as the N:S ratio decreases from 200:0 to 10:1, and then remains fairly constant at lower ratios. It is important to note that the differences between trials could also reflect uncontrolled differences in the milling process between the two sets of samples.

### Modeling Free Asparagine Content from Plant and Grain Measurements

**Figure 2:**
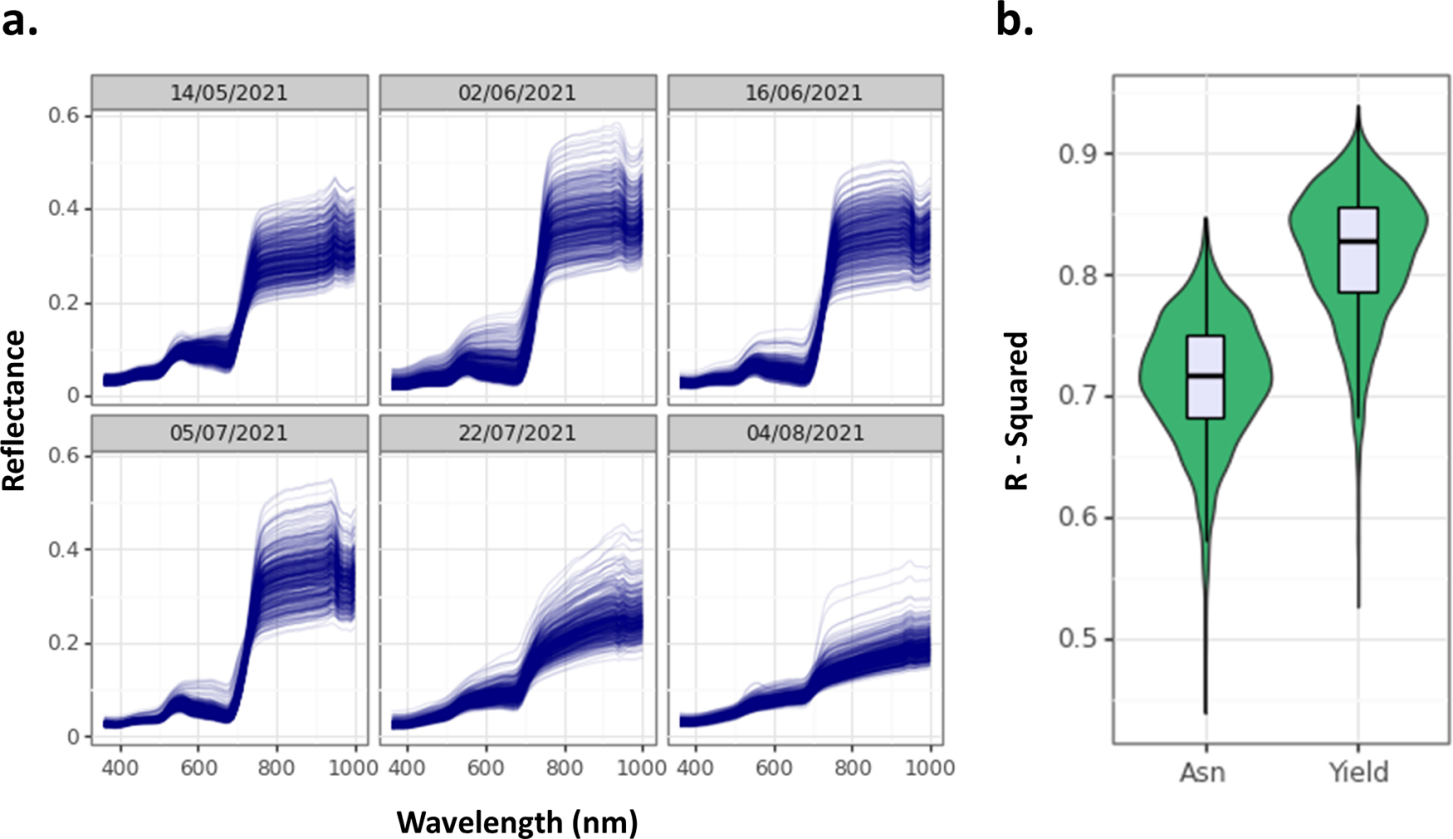
(a) Multispectral measurements taken from field plots for trial H21. (b) *R*^2^ values from partial least squares regression analysis of the data for free asparagine (Asn) and yield.

In trial H21, we collected multispectral measurements in the field for all 432 plots at six different timepoints until harvest (Figure 2a). Normalized difference vegetation index (NDVI) values calculated from these data showed differences between treatments, with plots lacking sulfur generally having lower NDVI values (Supplementary Figure 9). In order to test whether these data could be used to predict the free asparagine content of grain, we constructed partial least squares regression (PLSR) models using data from all six timepoints and tested the accuracy of this method for modeling grain free asparagine content and yield (Figure 2b). The model for free asparagine had an average *R*^2^ value of 71.26%, whereas the model for yield had an average *R*^2^ value of 81.75%. We also tested a classification model using Gaussian naïve Bayes to see whether these measurements could distinguish between sulfur-deficient (S0) and sulfur-fed (S10, S20, or S40) plots (Supplementary Figure 10). This model had a mean balanced accuracy of 0.76 (improvement of 0.26 over random classification).

**Figure 3:**
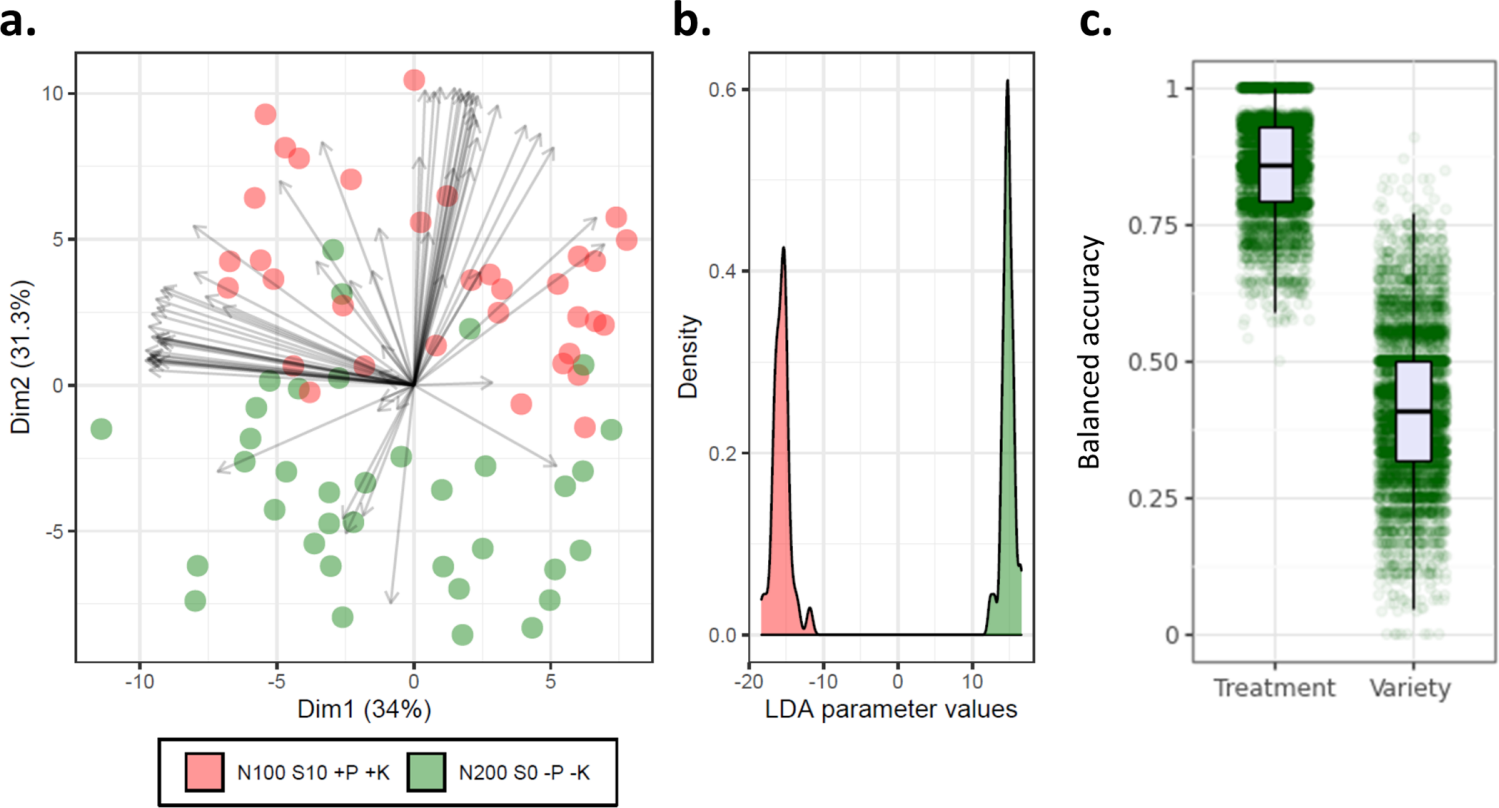
Measurements of selected seeds from trial H21. (a) Principal component analysis of all measured traits from Videometer SeedLab and grain asparagine content. (b) Linear discriminant analysis of seeds separated by agronomic treatment. (c) Balanced accuracy scores from Gaussian naïve Bayes classification for sample treatment and variety. −P (0 kg/ha phosphorus), −K (0 kg/ha potassium), +P (35 kg/ha phosphorus), and +K (62 kg/ha potassium).

Following on from this experiment, we investigated whether multispectral, fluorescence, and morphology measurements from the seed itself could be used to distinguish high asparagine seeds (>10 mmol/kg, from treatment N200 S0 −P −K) from low asparagine seeds (<5 mmol/kg, from treatment N100 S10 +P +K). Seeds from different agronomic treatments did tend to separate out along the second principal component from our PCA (Figure 3a), indicating that a classification model may be effective. We then performed a linear discriminant analysis, which showed good separation of treatments (Figure 3b) and tested the accuracy of a Gaussian naïve Bayes classifier using 1000 repeated five-fold cross validation (Figure 3c). This model was able to classify samples to the correct agronomic treatment group with a balanced accuracy of 0.86 (improvement of 0.36 over random classification) and was able to classify samples to the correct variety group with a balanced accuracy of 0.42 (improvement of 0.34 over random classification).

### Impact of Different Agronomic Treatments on Biscuit Quality and Acrylamide Formation

**Figure 4:**
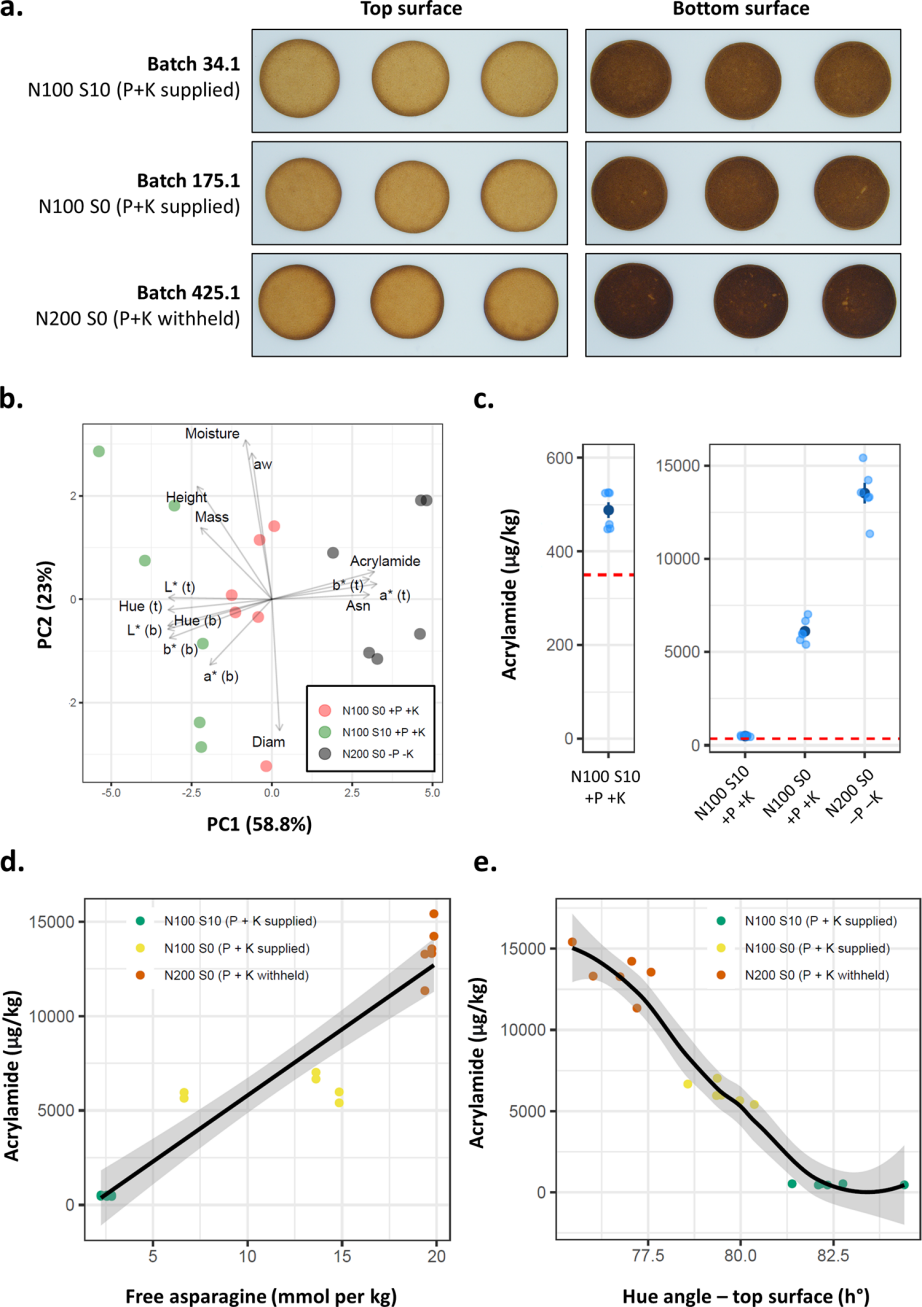
Acrylamide measurements in biscuits and associations with other variables. (a) Representative images of biscuits baked in this study. (b) PCA of all measurements taken from biscuits. (c) Acrylamide concentration of biscuits produced from grain from different agronomic treatments. The EU benchmark value of 350 μg/kg is given by the dashed red line. Dark blue points and bars show mean and standard error of the means, respectively. This plot is split to better visualize the lowest acrylamide concentrations of the S10 treatment. (d) Association between the free asparagine content of grain from plots selected for baking and biscuit acrylamide content. Linear model line fitted. (e) Association between biscuit color (as measured by hue angle of the top surface) and acrylamide content. LOWESS smoothing curve fitted. Gray-shaded region indicates the standard error of the means. −P (0 kg/ha phosphorus), −K (0 kg/ha potassium), +P (35 kg/ha phosphorus), +K (62 kg/ha potassium), t (top side of biscuit), and b (bottom side of biscuit).

In order to assess the impact of different agronomic treatments on end products, we baked biscuits from selected flours in the H21 trial (Figure 4a). We chose to bake biscuits using flour from variety Basset in three different agronomic treatments (N100 S10 +P +K, N100 S0 +P +K, and N200 S0 −P −K) because of the range in grain asparagine content shown by these samples. Biscuits baked from these different agronomic treatments tended to separate along the first principal component in the principal component analysis (Figure 4b), largely due to differences in acrylamide content, grain asparagine content, and color. The groups did not separate out along the second principal component, which mostly highlighted differences in moisture content and diameter.

Acrylamide content varied widely between the three agronomic groups, with all samples exceeding the EU benchmark level of 350 μg/kg for biscuits (Figure 4c). The biscuits from the N100 S10 +P +K treatment group contained 488 μg/kg mean acrylamide, and the N100 S0 +P +K and N200 S0 −P −K treatment groups contained 6114 and 13,523 μg/kg mean acrylamide, respectively. These differences were significant between all treatment groups (one-way ANOVA and Tukey tests, *p* < 0.001). Biscuit acrylamide content did correlate with free asparagine content of the grain (Figure 4d) and the top surface hue angle (Figure 4e), with Kendall correlations of 0.79 and −0.79 and *R*^2^ values from linear models of 89 and 91%, respectively (*p* < 0.001).

## Discussion

### Optimizing Fertilizer Application to Reduce Free Asparagine Content of Wheat Grain

Our results indicate that the application of nitrogen (N) and sulfur (S) at a ratio of 10:1 is sufficient to prevent large increases in asparagine accumulation in the grain of wheat grown on loamy sand/sandy loam soils. Previous studies investigating a range of S application rates have recommended that 20 kg/ha S should be applied (equivalent to 50 kg/ha SO_3_).^45^ Our findings agree that at higher application rates of N (200 kg/ha), 20 kg/ha S application is required to minimize asparagine accumulation. However, at lower rates of N application (100 kg/ha), 10 kg/ha S application is sufficient. Application rates greater than a 10:1 N to S ratio did not contribute to any meaningful further reduction in free asparagine content in the grain, so application above this rate should be carefully considered due to the potential negative effects of S over-application on the environment.^46^ The average field rate for N application on winter wheat in the UK in 2021 was 188 kg/ha, while the average S application rate was 20.8 kg/ha (equivalent to 52 kg/ha SO_3_),^47^ equivalent to a mean N:S ratio of approximately 9:1. However, while N was applied to 99% of the winter wheat area, S was applied to only 73%, meaning that 27% of the winter wheat area in 2021 received high levels of N without any S. Different soil types will require different application rates of N and S due to differences in endogenous nutrient content, so further testing on sites with different soil profiles should be undertaken, and some of the wheat not receiving S may be used for feed or bioenergy. However, this does suggest that persuading farmers who are currently not applying S to their winter wheat to do so would facilitate regulatory compliance on acrylamide for food businesses and reduce the exposure of consumers to acrylamide from wheat products. S application also improves a number of other desirable traits, which we did not measure in our study.^48^

Interestingly, we found that withholding potassium and phosphorus application did not cause increases in free asparagine content in the grain from either trial when S was applied at 20 kg/ha, but we did observe an increase in the sulfur-deficient plots in the second trial when phosphorus and potassium were absent. Previous studies have shown that potassium and phosphorus deficiencies cause increases in asparagine in the root, stem, and leaves of a range of plant species,^49,50^ so we thought we might observe a similar increase in wheat grain regardless of S application, but this was not the case. Our study did not detect an overall effect of phosphorus and/or potassium application on yield either, suggesting that phosphorus and potassium may already have been present in sufficient concentrations in the soil at both trial sites, despite the soil type. This further emphasizes the need to test soil nutrient content and tailor fertilizer application so that only the required amount is applied.^9^ Nevertheless, our observation that phosphorus and potassium deficiencies may cause increases in asparagine content in grain during S deficiency warrants further study.

In addition to different N:S ratios, free asparagine content of grain also differed between the different varieties used in this study. Varietal differences in free asparagine in wheat grain have been observed in many studies, but they are often much smaller than the differences associated with environmental factors.^51,52^ In this study, the differences between varieties were also much smaller than the differences between fertilizer treatments. The same pattern has been observed between varieties that do and do not possess the *TaASN-B2* gene: while there were differences in free asparagine content in the grain between such varieties, much larger differences were again caused by S deficiency.^26^ Consequently, the use of varieties that are lower in free asparagine content in the grain will only be effective if a low N:S ratio is maintained. Sulfur deficiency also impacts on other desirable traits,^48^ and our overall recommendation is for farmers to focus on applying nitrogen and sulfur at a ratio of 10:1 kg/ha.

### Modeling the Free Asparagine Content of Grain Using Imaging Technology

Burnett et al.^53^ demonstrated for the first time that hyperspectral imaging of plants can be used to effectively predict certain metabolites produced during stress (in their study, abscisic acid and proline were analyzed during drought stress) and outlined how such analyses can be performed.^54^ Similarly, we found that multispectral measurements of wheat grown in the field were able to predict grain asparagine content with an average accuracy of 71% when used in our PLSR model. The screening efficacy of our model is likely due to the dual impact of sulfur deficiency on wheat canopy color (sulfur deficiency caused a yellowing of the canopy, shown by our NDVI and PSRI measurements) and grain asparagine content. Few studies have investigated sulfur deficiency through multispectral imaging, as most studies of this sort have focused on nitrogen,^55^ but Mahajan et al.^56^ found that certain vegetation indices could predict sulfur content in wheat with an accuracy of 0.46. Further development of these models with independent prediction and validation sets will be valuable for testing whether multispectral measurements from the field can be used to accurately predict grain asparagine content and sulfur deficiency.

Our model for classification of seeds was also able to distinguish sulfur-deficient from sulfur-fed samples. Models such as these, if developed appropriately, could be useful for millers to quickly screen grain samples to determine grain quality. Classification of wheat seeds using spectroscopy can be used for traits such as protein content, Hagberg falling number, and pathogen damage,^23^ so prediction of asparagine content could be integrated into such models to give an overall measurement of grain quality. However, for models on both plants and seeds to have broad applicability, they would need to be trained using samples from many more diverse environments and under diverse stressors. Many other stressors are associated with grain asparagine accumulation,^25^ and spectroscopy can be used to measure such stressors,^57^ so future experiments should investigate the accuracy of these models under more stressors.

### Impact of Different Treatments on Biscuit Quality

Flours from different agronomic treatments differed greatly in terms of acrylamide content in this study, and there was a strong correlation between asparagine and acrylamide, showing that agronomic strategies to control grain asparagine content can effectively control the acrylamide content of biscuits. There was also a strong correlation between color (hue angle) and acrylamide content, as observed previously in biscuits,^58^ indicating that biscuit color can also be used to predict acrylamide concentration. In-line color sorting could therefore be implemented on biscuit production lines to eliminate high acrylamide products, as has been recommended for other products.^18^ Acrylamide formation will differ in biscuits depending on differences in ingredients and processing as well, so it would be advisable to check the correlation between the asparagine content of the flour used and the end-product acrylamide concentration to ensure that strategies to reduce grain asparagine content will be effective to control acrylamide.

Interestingly, we found acrylamide concentrations exceeding the benchmark level for biscuits (350 μg/kg) even in those samples where asparagine concentration was low (2–3 mmol/kg). This is likely due to the baking methodology and oven used in this study. For example, our recipe included ammonium bicarbonate and high-fructose corn syrup, the combination of which is known to greatly elevate acrylamide formation compared to sodium bicarbonate and glucose.^59^ The oven used in this study was also not a conventional traveling oven used for baking biscuits, so heat transfer may have occurred more rapidly. Consequently, it is important to implement baking processes that do not favor acrylamide formation even when using low asparagine flours as unfavorable processing conditions can create products exceeding benchmark levels, even from flours that are relatively low risk.

## Supporting Information Available

Additional tables (Supplementary Tables 1 and 2) and figures (Supplementary Figures 1−10) concerning experimental design, differences in asparagine content between treatments, and differences in multispectral imaging measurements (PDF)
Detailed description of fertilizer treatments used in the two trials (XLSX)
Imaging data collected from grain samples using the Videometer SeedLab system (XLSX)
Complete ANOVA tables for analyses performed in this study investigating the overall impact of treatment (XLSX)
Complete ANOVA tables for analyses performed in this study investigating the individual impact of nutrients (nitrogen, sulfur, potassium, and phosphorus) (XLSX)
Grain asparagine content data from samples from both trials (XLSX)
Multispectral reflectance data from the second trial across all measured timepoints (XLSX)
Acrylamide and other measurement data taken from biscuits baked in this study (XLSX)
