# Supplementary material for: Reducing Dietary Acrylamide Exposure from Wheat Products through Crop Management and Imaging
Source: J Agric Food Chem. 2023 Feb 6;71(7):3403–13. doi: 10.1021/acs.jafc.2c07208 (PMC9951245; doi:10.1021/acs.jafc.2c07208)
Supplement: Supplementary file 1 — jf2c07208_si_001.pdf [file jf2c07208_si_001.pdf]

Supporting information

Reducing dietary acrylamide exposure from wheat products through crop management and imaging

Joseph Oddy[†], John Addy[†], Andrew Mead[†], Chris Hall[†], Chris Mackay[†], Tom Ashfield[†,‡], Faye McDiarmid[‡], Tanya Y. Curtis[§], Sarah Raffan[†], Mark Wilkinson[†], J. Stephen Elmore[#], Nicholas Cryer[⊥], Isabel Moreira de Almeida[▴], and Nigel G. Halford[†*]

[†]Rothamsted Research, Harpenden, Hertfordshire AL5 2JQ, United Kingdom, [‡]Crop Health and Protection (CHAP), Rothamsted Research, Harpenden AL5 2JQ, United Kingdom, [§]Curtis Analytics Limited, Discovery Park, Sandwich, United Kingdom, [#]Department of Food & Nutritional Sciences, University of Reading, RG6 6DZ, UK
[⊥]Mondelēz UK R&D Ltd, Bournville Lane, Bournville, Birmingham, B30 2LU, UK
[▴]Mondelēz R&D International, 6 Rue René Razel, 91400 Saclay, France

* Author to whom correspondence should be addressed (*E-mail nigel.halford@rothamsted.ac.uk).*

Supplementary Table 1. Key dates in the two trials undertaken in this study.

Trial	Drilling	First split	Second split	Harvest
Stackyard	04/12/2019	10/03/2020	01/06/2020	10-11/09/2020
Butt Clong	26/11/2020	23/02/2021	17/05/2021	20/08/2021

Supplementary Table 2. Significance values (F probabilities) of terms in Nitrogen/Sulphur ANOVA models for analysis of Log_e transformed grain asparagine content.

Source of variation	H20	H21	Both
Nitrogen	0.078	<.001	0.005
Sulphur	0.028	<.001	<.001
Nitrogen*Sulphur	0.987	0.015	0.664
Variety	<.001	<.001	<.001
Nitrogen*Variety	0.133	0.166	0.178
Sulphur*Variety	0.773	0.026	0.684
Nitrogen*Sulphur*Variety	0.069	0.050	0.067
Trial	NA	NA	0.012
Trial*Nitrogen	NA	NA	0.673
Trial*Sulphur	NA	NA	0.245
Trial*Nitrogen*Sulphur	NA	NA	0.935
Trial*Variety	NA	NA	<.001
Trial*Nitrogen*Variety	NA	NA	0.052
Trial*Sulphur*Variety	NA	NA	0.086
Trial*Nitrogen*Sulphur*Variety	NA	NA	0.462

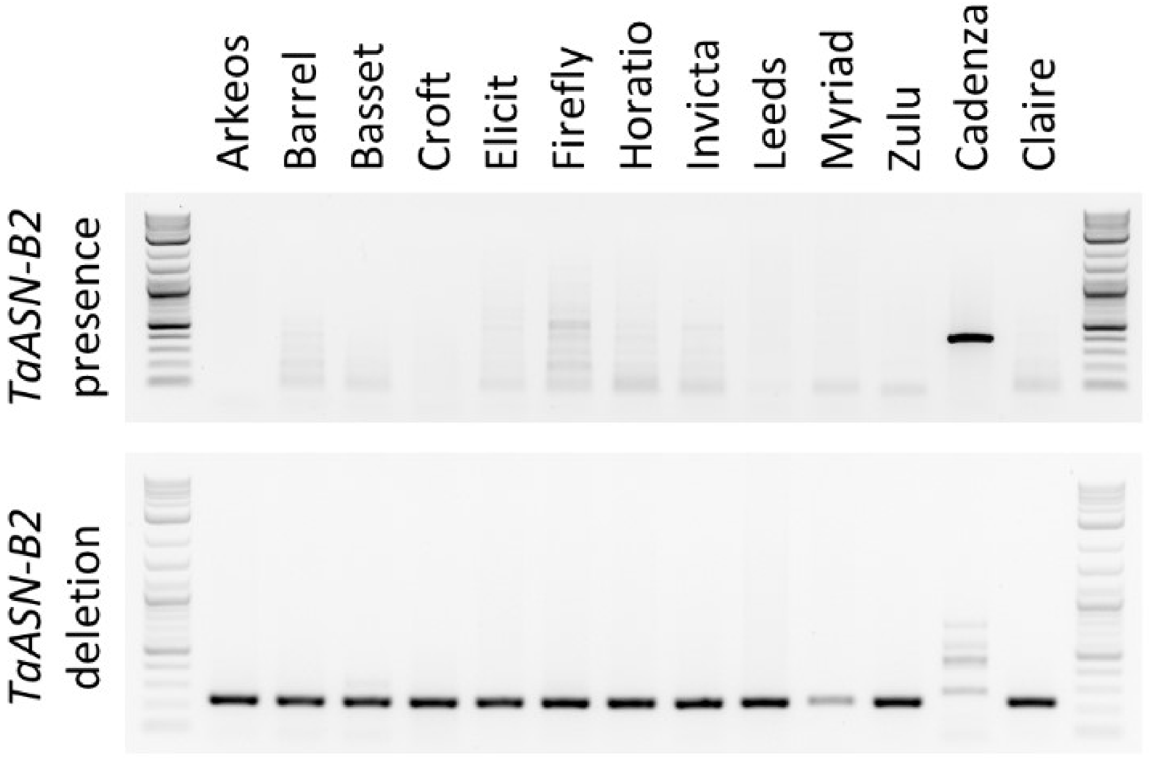

Supplementary Figure 1. Screening for the presence/absence of the *TaASN-B2* gene in the soft wheat varieties analysed in this study. Cadenza was used as a positive control and Claire as a negative control.

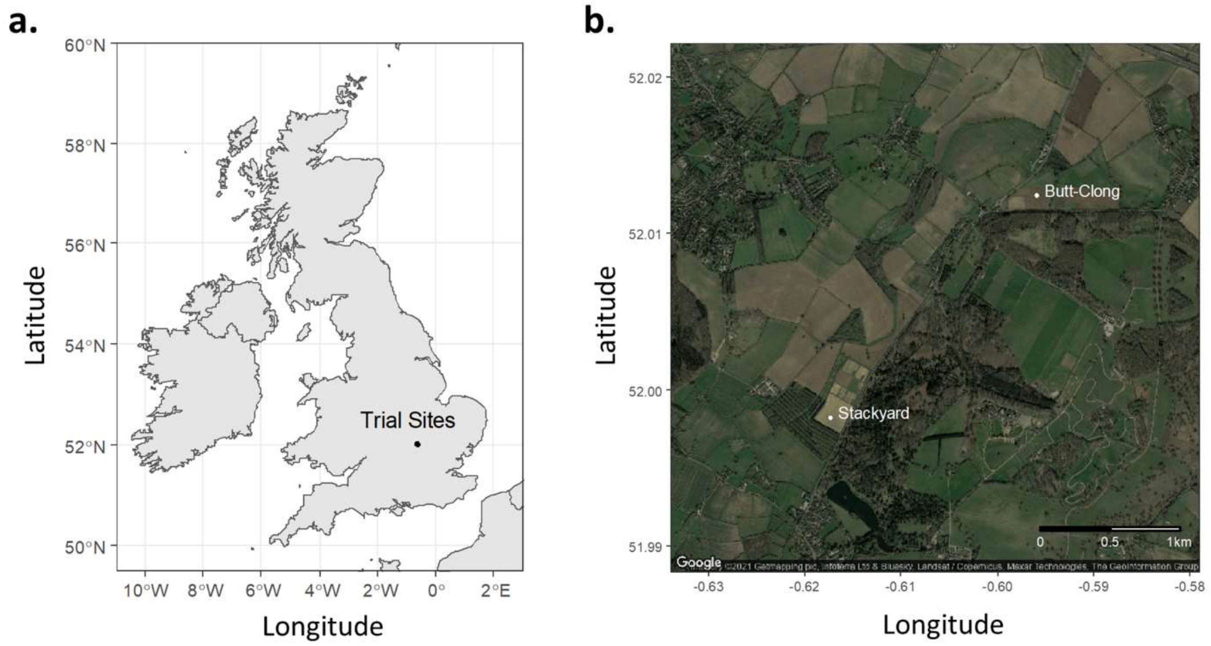

Supplementary Figure 2. Location of the trial sites relative to the British Isles (**a.**) and at finer scale (**b.**).

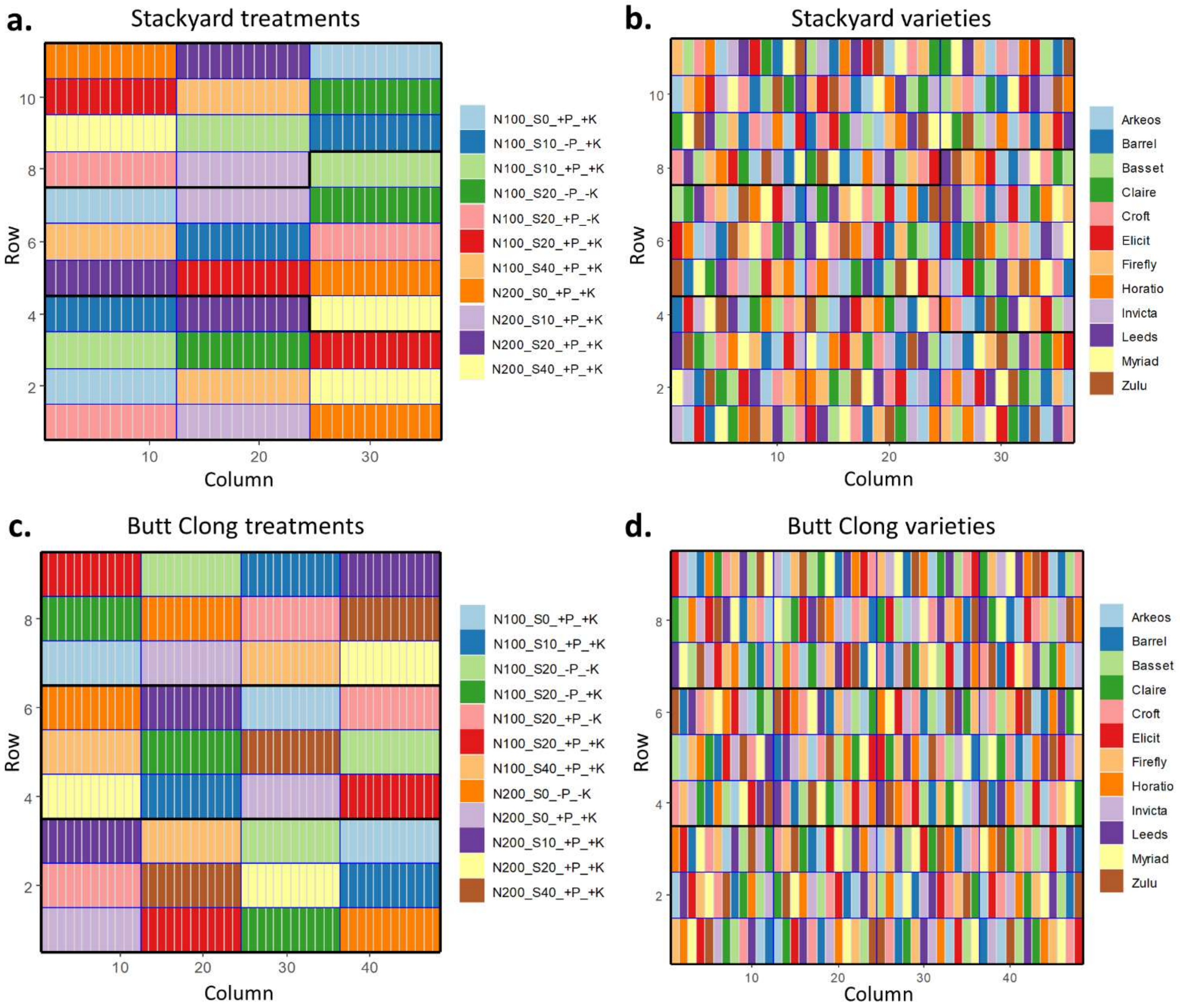

Supplementary Figure 3. Layout of the treatments and varieties in Stackyard in 2019 – 2020 (**a.** and **b.**) and in Butt Clong in 2020 – 2021 (**c.** and **d.**).

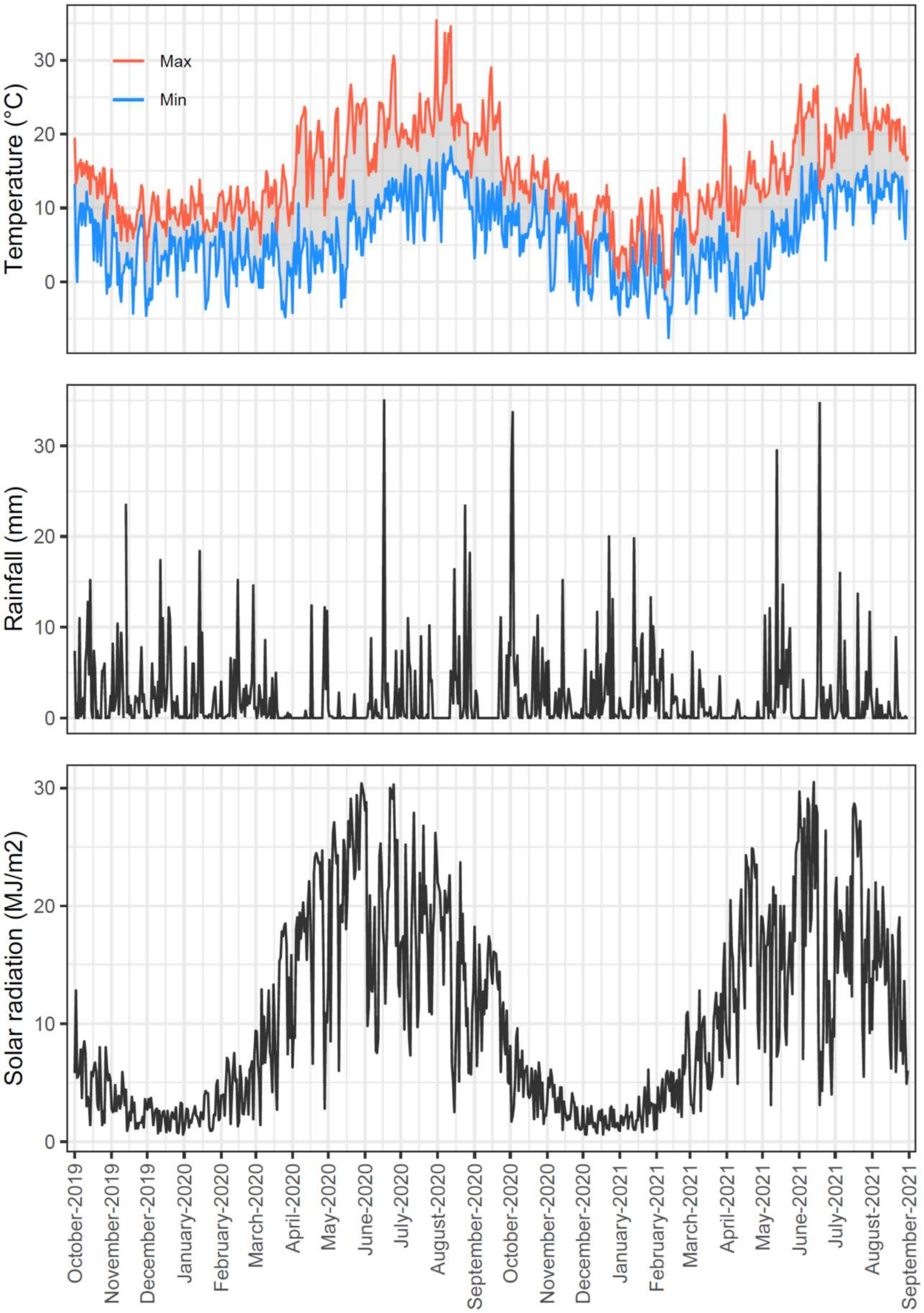

Supplementary Figure 4. Temperature, rainfall, and solar radiation measurements during both field trials measured at the Woburn weather station.

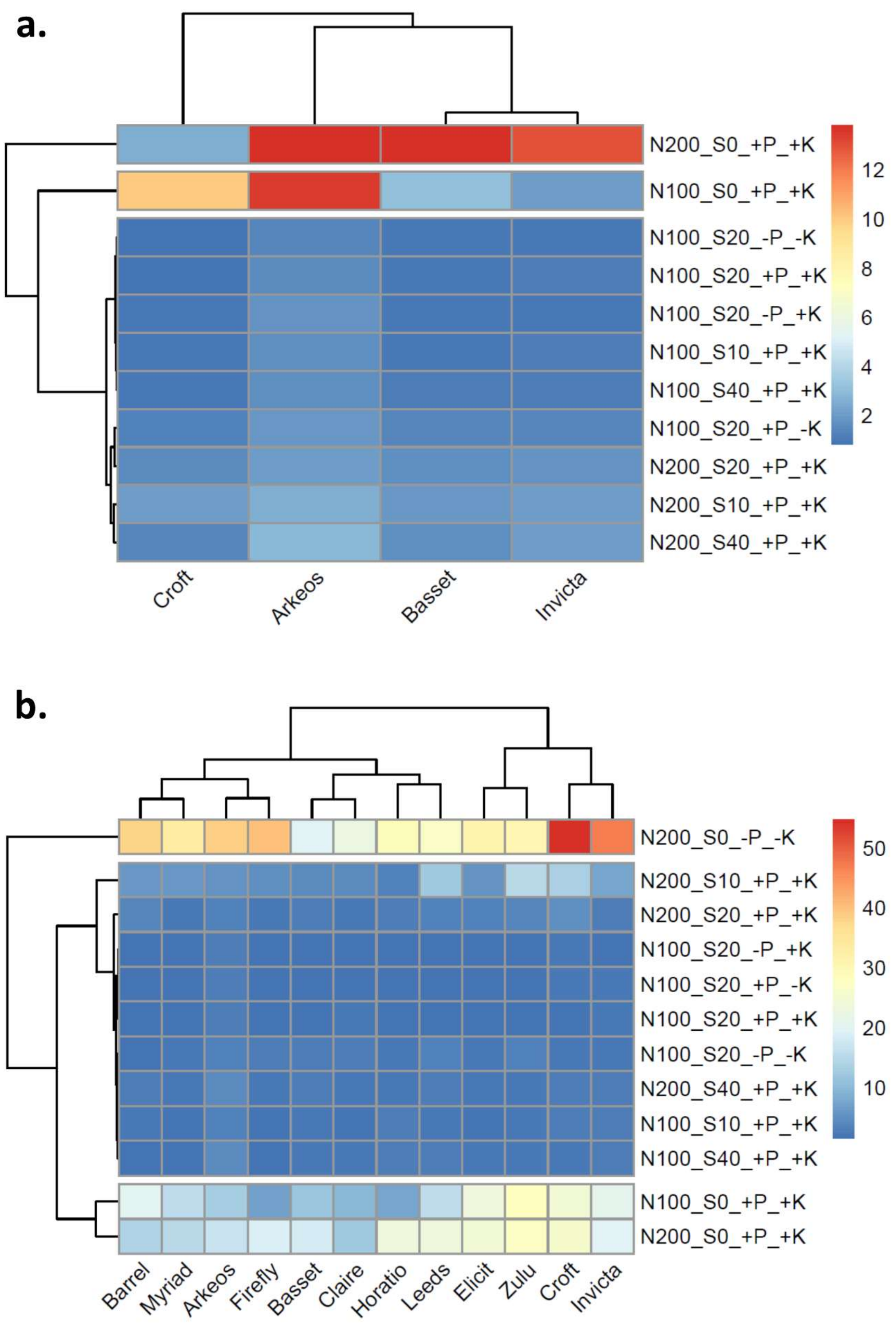

Supplementary Figure 5. Non-transformed mean asparagine content in trial H20 (**a.**) and H21 (**b.**) for each variety and treatment combination.

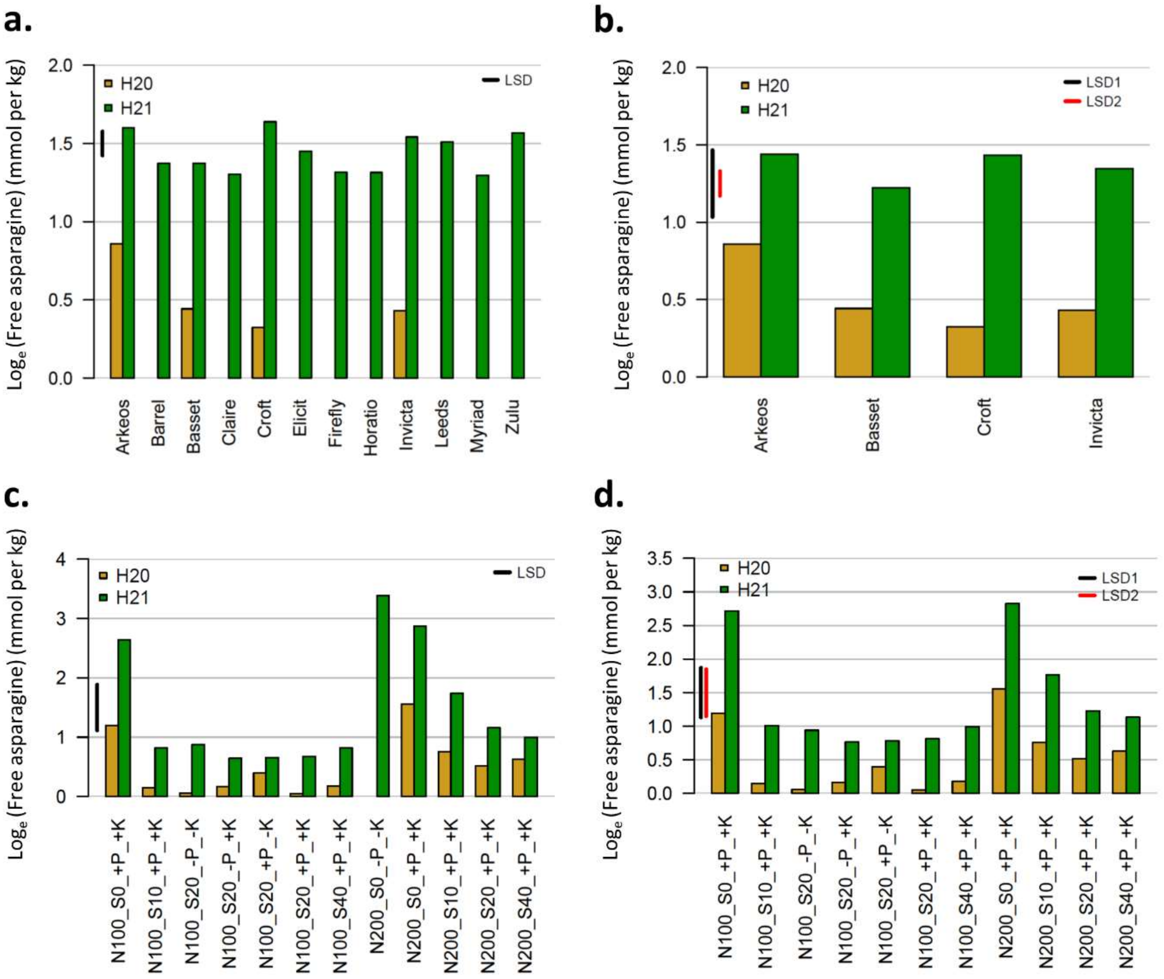

Supplementary Figure 6. Mean grain asparagine content for each variety and fertiliser treatment in both trials and in different analyses. **a.** Variety means from nested model. **b.** Variety means from full model. **c.** Treatment means from nested model. **d.** Treatment means from full model. N (nitrogen), S (sulphur), P (phosphorus), K (potassium). Fertiliser application rates for N and S given in kg per hectare. Nested model LSDs (least significant differences) set at 5% and only valid for comparisons within each trial. Full model LSDs (least significant differences) set at 5%. LSD1s valid for comparison between trials and treatments/varieties. LSD2s only valid when comparing same level of trial.

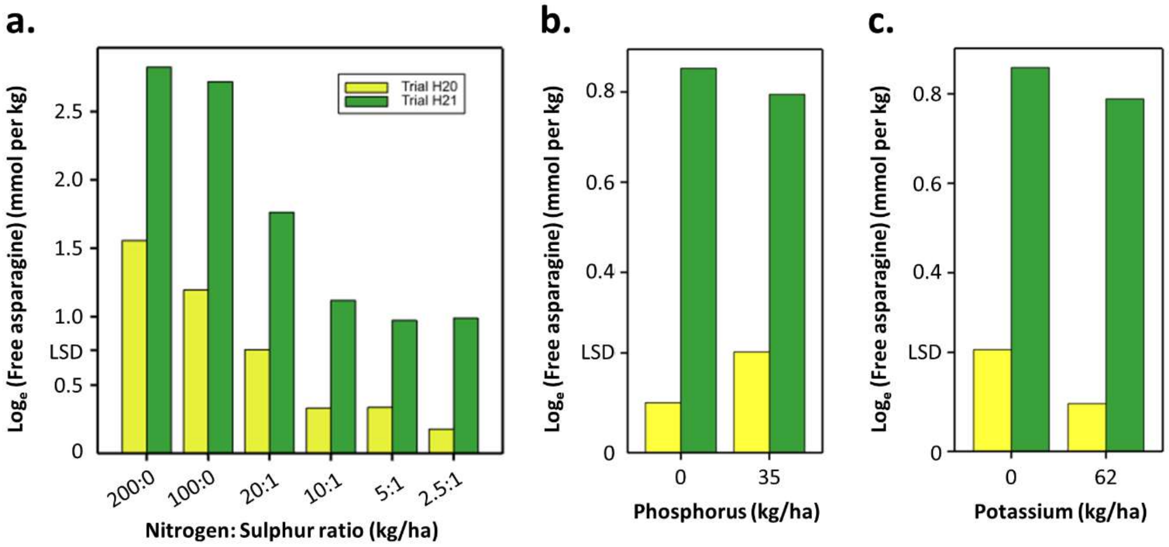

Supplementary Figure 7. Mean grain asparagine content at different nitrogen: sulphur ratios (**a.**) and in the presence or absence of phosphorus (**b.**) and potassium (**c.**) fertiliser. LSDs valid for comparison between trials and treatments.

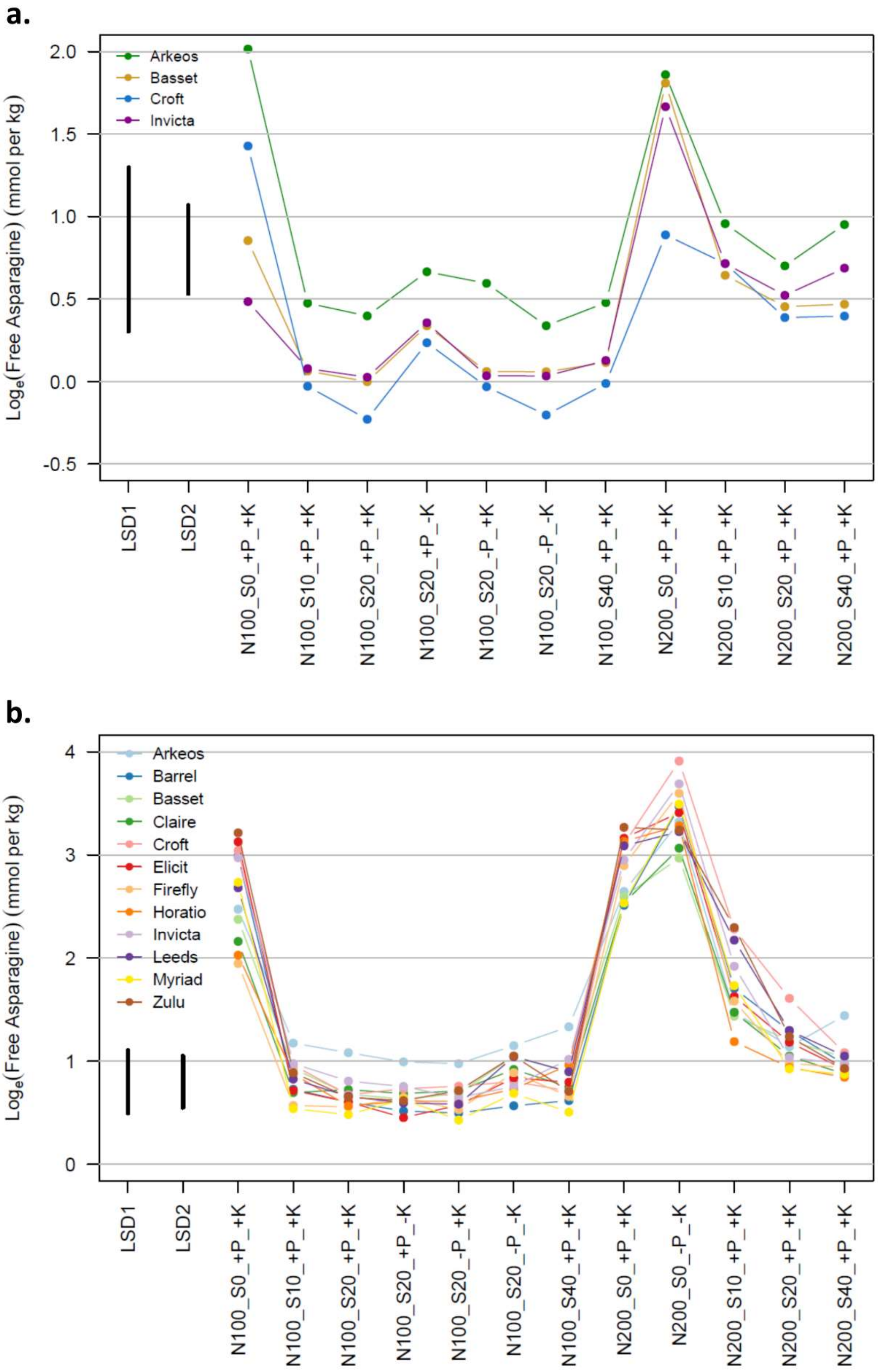

Supplementary Figure 8. Mean $\log_e$ transformed asparagine content in trials H20 (**a.**) and H21 (**b.**) for each variety and treatment combination.

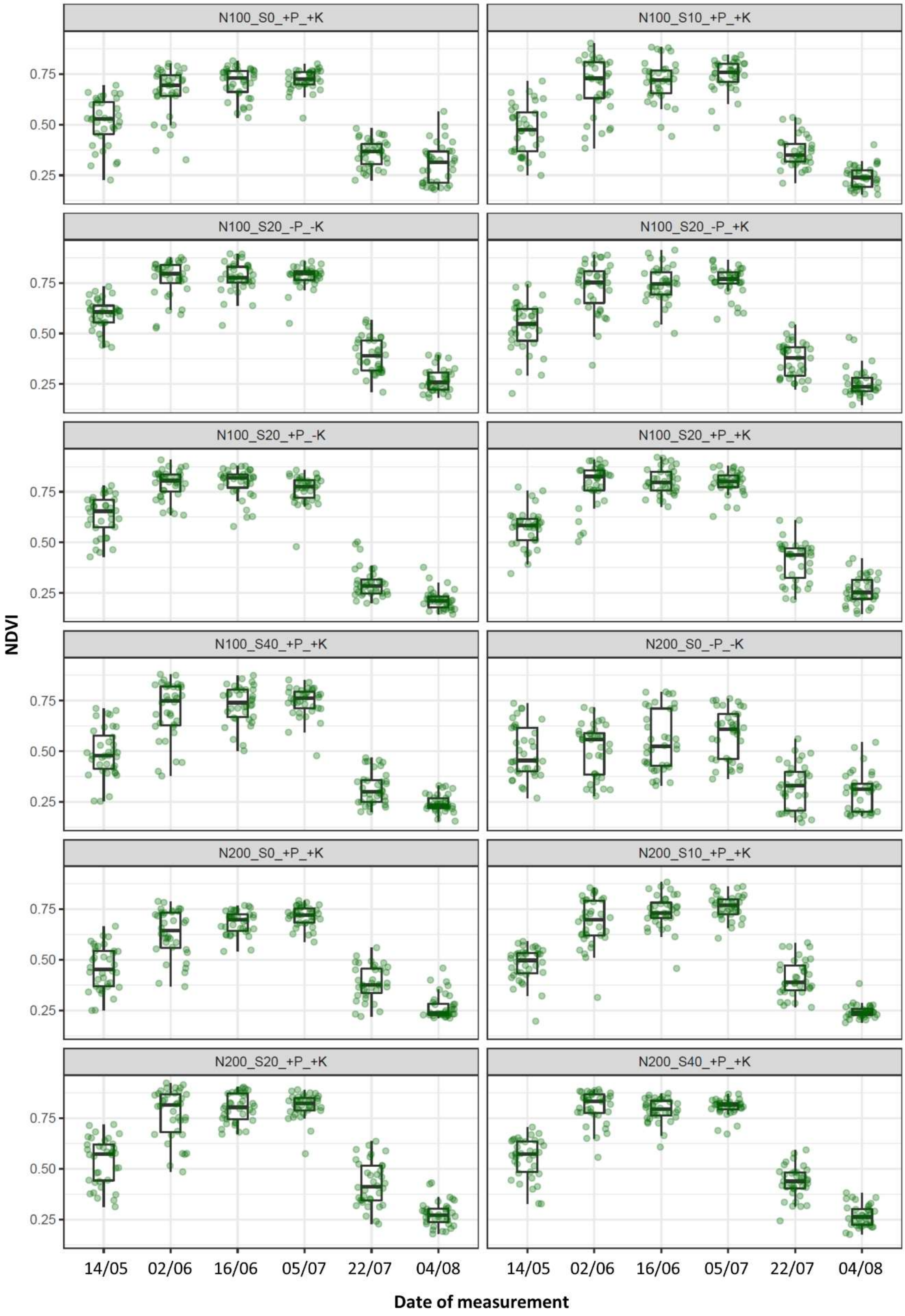

Supplementary Figure 9. Normalised difference vegetation index measurements for all plots over all six measurement dates separated by agronomic treatment.

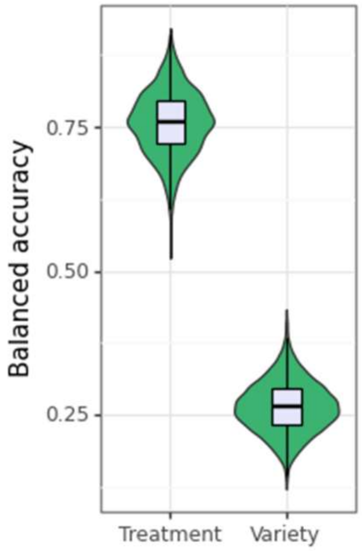

Supplementary Figure 10. Balanced accuracy values from gaussian naïve Bayes classification models distinguishing sulphur deficient vs. sulphur fed plots (Treatment) and varieties using multispectral data from the H21 field trial.
